# *Cryptosporidium* and Colon Cancer: Cause or Consequence?

**DOI:** 10.3390/microorganisms8111665

**Published:** 2020-10-27

**Authors:** Manasi Sawant, Martha Baydoun, Colette Creusy, Magali Chabé, Eric Viscogliosi, Gabriela Certad, Sadia Benamrouz-Vanneste

**Affiliations:** 1 Université de Lille, CNRS, INSERM, CHU Lille, Institut Pasteur de Lille, U1019-UMR 9017-CIIL-Centre d’Infection et d’Immunité de Lille, F-59000 Lille, France; manasi.sawant@pasteur-lille.fr (M.S.); martha.baydoun@ibl.cnrs.fr (M.B.); magali.chabe@univ-lille.fr (M.C.); eric.viscogliosi@pasteur-lille.fr (E.V.); sadia.benamrouz@univ-catholille.fr (S.B.-V.); 2 INSERM, CHU-Lille, U1189-ONCO-THAI-Assisted Laser Therapy and Immunotherapy for Oncology, Université de Lille, F-59000 Lille, France; 3 Service d’Anatomie et de Cytologie Pathologiques, Groupement des Hôpitaux de l’Institut Catholique de Lille, F-59000 Lille, France; creusy.colette@ghicl.net; 4 Faculté de Pharmacie, Université de Lille, F-59000 Lille, France; 5 Délégation à la Recherche Clinique et à l’Innovation, Groupement des Hôpitaux de l’Institut Catholique de Lille, F-59462 Lomme, France; 6 Equipe Ecologie et Biodiversité, Unité de Recherche Smart and Sustainable Cities, Faculté de Gestion, Economie et Sciences, Institut Catholique de Lille, F-59000 Lille, France

**Keywords:** *Cryptosporidium* infection, infection and cancer, digestive cancer, colon cancer, carcinogenesis

## Abstract

The number of cancers attributable to infectious agents represents over 20% of the global cancer burden. The apicomplexan intracellular parasite *Cryptosporidium* is currently considered one of the major causes of mild and severe diarrhea worldwide. However, less attention has been paid to its tumorigenic potential despite the high exposure of humans and animals to this ubiquitous parasite. Herein, we discuss the potential causal link between *Cryptosporidium* infection and digestive cancer, with particular emphasis on colon cancer, based on increasing clinical, epidemiological and experimental pieces of evidence supporting this association. In addition, we highlight the current knowledge about the potential mechanisms by which this parasite may contribute to cell transformation and parasite-induced cancer.

## 1. Introduction

One of the biggest obstacles to increase life expectancy in the 21st century is cancer since this disease causes about 13% of human mortality [1]. Behind the strategy of treatment and early detection, it is important to work on cancer prevention, considering that between 40% and 45% of cancers are associated with preventable risk factors, including tobacco smoke, lack of physical activity, obesity, dietary factors, exposition to solar ultraviolet (UV) radiation or infectious agents [2,3]. Subsequently, reducing the burden of cancer is possible if these risk factors could be identified and if population exposure to them could be avoided or at least reduced [3]. In particular, the role of some infectious agents as carcinogens has been already recognized by the International Agency for Research on Cancer (IARC). However, additional pathogens are probably involved in specific human cancers. This review will thus be focused on the causal link between infection and cancer, including an update on the association between the infection by the protozoan *Cryptosporidium* and digestive cancer. Besides the several experimental and epidemiological studies that have revealed this link, mechanistic studies have shown that this parasite is able to hijack the host-cell machinery, potentially leading to a transformation of the host cell.

## 2. Infection, an Important Cause of Cancer

Causal associations between infectious agents and the development of human cancers have already been highlighted [4]. Overall, the total number of cancer attributable to infectious agents in 2002 was estimated at 1.9 million cases, standing for 17.8% of the global cancer burden. Moreover, it has been hypothesized that, by 2050, the majority of human cancers could be due to infections [5,6]. However, proving that infectious agents are causative factors of human cancer remains difficult for many reasons, such as:

- The periods between primary infection and malignant transformation are frequently very long [7].
- Even if the majority of the infectious agents associated to human cancers are ubiquitous and common in the human population, only a small proportion of infected individuals develops cancer.
- Some infections are linked to cancer development as associated risk factors [8].
- Infectious agents act mainly as indirect oncogenes, without persistence of their genes within the respective cancer host cells. The most common indirect infectious carcinogens are agents causing immunosuppression, such as Human Immunodeficiency Virus (HIV) leading to Kaposi’s sarcoma, or inflammation caused by the bacteria *Helicobater pylori*, the trematode *Schistosoma hematobium* and the Hepatitis C and B viruses [7].
- The main mechanisms by which infectious agents promote cancer are not necessarily involving direct mutagenesis, but instead are due to the complex interactions between hosts and pathogens [8].
- An infectious agent may trigger the initial events of oncogenesis while being absent in the final tumor [7].
- Pathogens associated with cancer are directing pathogen-driven processes leading to cell transformation. However, many non-oncogenic pathogens can also regulate these processes, indicating that other factors must be involved [8].
- In the cases of viruses, oncogenesis can occur through the persistence of the viral genome in a latent form in an infected host cell, either without replication or through integration of the viral genome into a host-cell chromosome [8].
- Koch’s postulates for proving a causal connection between a particular infectious agent and a disease cannot be applied to many human diseases as it would be unethical to experimentally infect humans with a potentially lethal infectious agent [8].
- Existing diagnostic tools may not be sensitive enough to link infectious agents with cancer development or testing may occur too long after the exposure [9].

**Figure 1:**
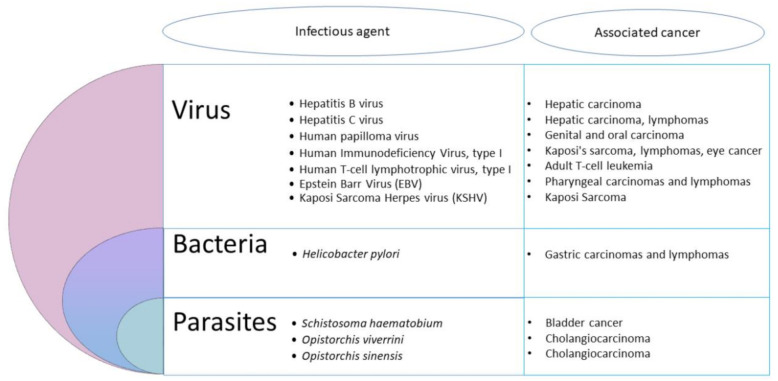
Infectious agents recognized by the International Agency for Research on Cancer (IARC) as major contributors to the global number of cancers.

Nevertheless, at least 11 biological agents have presently been recognized by the IARC as major contributors to the global number of cancers in humans (Figure 1).

These agents include viruses, bacteria and helminths. The most important infectious agents worldwide are *H. pylori* (5.5% of all cancer), the human papilloma viruses (HPVs) (5.2%), the hepatitis B and C viruses (4.9%), the Epstein–Barr virus (EBV) (1%), the HIV together with the human herpes virus 8 (0.9%) and the human T-cell leukemia/lymphoma virus type 1 (HTLV-I) (0.03%) [10]. Other pathogens, including parasites, are also considered carcinogenic agents in human beings. Among helminths, the widespread digenetic trematode *S. haematobium* has been associated to urinary bladder cancer, and the flukes *Opisthorchis viverrini* and *Clonorchis sinensis* are causally linked with cholangiocarcinoma [10]. So, the idea of parasites as a cause of cancer in vertebrates is slowly developing [11]. However, the contribution of intracellular eukaryotic parasites to cancer development has been largely neglected until now [2]. Yet, based on clinical and epidemiological pieces of evidence, many reports underlined a potential association between parasitic protozoan infections and cancer. Hence, the flagellate *Trichomonas vaginalis* was suspected to be associated with prostate [12] and cervical cancers [13], while the apicomplexan *Toxoplasma gondii* was suggested to be linked with ocular tumor, meningioma, leukemia and lymphomas [14]. It was also suggested that *Plasmodium falciparum* could play a co-factor role in the development of Burkitt lymphoma [14]. Nevertheless, only the apicomplexan genera *Cryptosporidium* and *Theileria* have been shown to induce cell transformation experimentally [2].

Pathogens use several strategies to target cellular processes during their parasitic interactions with the host cell. The identification of microbial proteins manipulating host functions to promote infection, proliferation and escape defenses has led to great progress in understanding the host’s cellular processes. Continued persistent infection by a pathogen requires host-cell survival, host-cell proliferation, and evasion of the immune system by the pathogen. These pathogen-driven processes are achieved through various mechanisms that interfere with normal cell physiology. Alterations in these normally highly regulated pathways can lead to transforming events that have been described as the ‘hallmarks of cancer’ [5]. Carcinogenic pathogens are also able to target epigenetic mechanisms to divert the host cellular machinery [2]. Interestingly, recent mechanistic studies suggest that apicomplexan eukaryotic intracellular parasites are indeed capable of reproducing some mechanistic aspects of tumorigenesis leading to cancers, either by their infection alone, or by the combination of the parasitic infection with environmental factors [15,16]. There is a growing number of pharmacological studies analyzing the effect of anti-cancer molecules on parasitic diseases and vice versa [17]. Overall, infection seems to play a crucial role in the etiology of cancer. Actually, it was estimated that there would be 26.3% fewer cancers in developing countries and 7.7% in developed countries if cancers associated with infectious diseases were prevented [18].

## 3. The Special Case of *Cryptosporidium*: A Public Health Issue

*Cryptosporidium* is the agent of cryptosporidiosis, an infection resulting from the ingestion of parasite oocysts mainly through the consumption of fecally contaminated food or water, or through direct contact with the infected host [19,20]. *Cryptosporidium parvum* and *C. hominis* are the two species responsible for the majority of human cases of cryptosporidiosis. This parasite is considered a major cause of diarrhea worldwide. It causes self-limited watery diarrhea in immunocompetent individuals, but has devastating effects in those who are immunocompromised. In young children, malnutrition, growth and cognitive deficits were reported as sequels of cryptosporidiosis [21,22]. Most strikingly, a cohort study (GEMS) involving 22,500 children in Africa and Asia revealed that *Cryptosporidium* is one of the four main pathogens responsible for severe diarrhea and mortality in infants and toddlers [23]. This parasite was then considered the second leading cause of death in children due to diarrhea [24]. More recently, the Global Burden of Diseases, Injuries, and Risk Factors Study (GBD) analyzed in 2016 the burden of diarrhea in 195 countries and reported that *Cryptosporidium* is the fourth leading cause of diarrhea mortality among children under 5 years of age (with 48,301 annual death) [25,26]. It has also been reported that the substantial short-term burden of diarrhea induced by *Cryptosporidium* infection on childhood growth and well-being is largely underestimated [25,27] probably due to a significant proportion of asymptomatic or mild and self-limiting infections, which consequently remain not diagnosed. In addition, *Cryptosporidium* species are responsible for numerous waterborne outbreaks of gastrointestinal disease. The most extensive was described in 1993 in Milwaukee, USA, where over 400,000 people became ill (the population of this area was approximately 1.61 million) with 69 deaths [28,29]. The number of outbreaks caused by *Cryptosporidium* is increasing worldwide since 239 waterborne outbreaks were reported in Europe, Australia and North America between 2011 and 2016 versus only 120 in the same area between 2004 and 2010 [30,31]. The Centers for Disease Control and Prevention (CDC) also published that 32 outbreaks were caused by *Cryptosporidium* in the United States in 2016 and linked them to swimming pools and water playgrounds against only 13 in 2013 [32]. As a result, an ever-growing number of people would be exposed to this pathogen and not only in developing countries. Despite its prevalence and impact on public health, neither treatment nor vaccine against *Cryptosporidium* are yet available.

## 4. *Cryptosporidium* and Cancer: A Growing Body of Evidence

**Figure 2:**
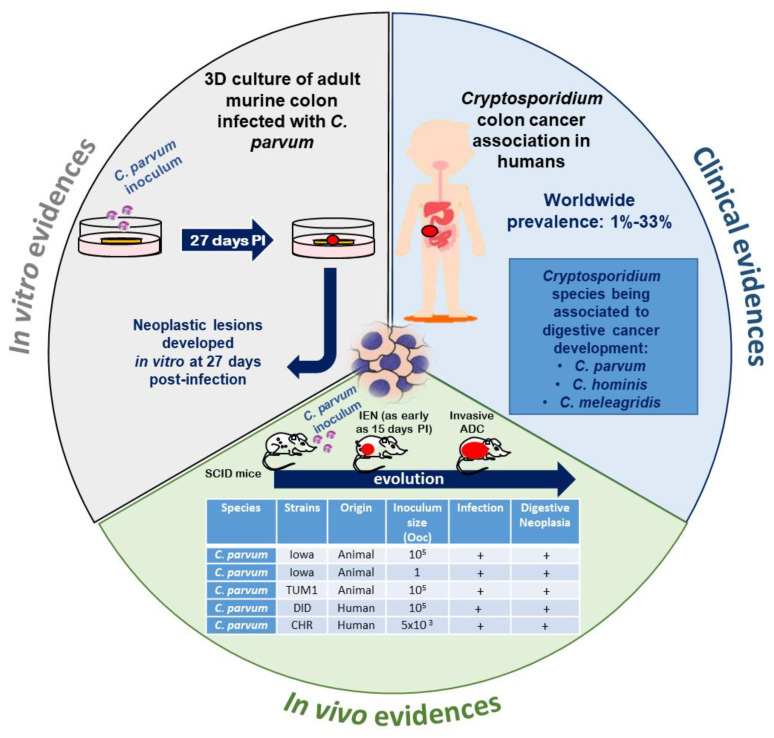
Recent experimental and clinical approaches contributing to expanding the understanding of the role of *Cryptosporidium* in the induction of digestive neoplasia. PI: post-infection, IEN: intraepithelial neoplasia, ADC: adenocarcinoma, Ooc: oocysts. Source of pictograms: https://fr.freepik.com/photos-vecteurs-libre/banner”>Banner vecteur créé par pch.vector—fr.freepik.com</a>.

In various animal groups and in humans, epidemiological and experimental studies tend to reinforce the hypothesis of an association between *Cryptosporidium* infection and cancer (Figure 2).

### 4.1. Clinical Studies in Humans

**Figure 3:**
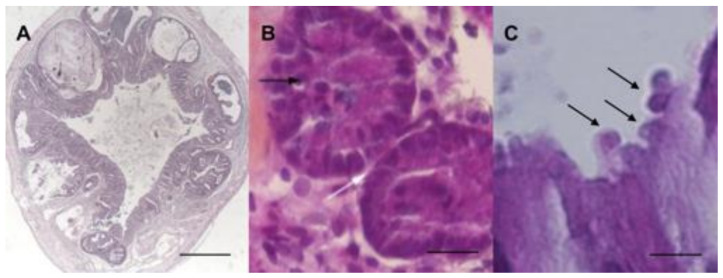
(**A**) Adenocarcinoma of the ileo-caecal region in a Dex-treated Severe Combined Immunodeficiency (SCID) mouse after 62 days of *C. parvum* infection. Scale bar, 1000 μm. (**B**) Low-grade intraepithelial neoplasia in a murine colonic explant after 27 days of *C. parvum* infection characterized by reduction of the interglandular space (white arrow), and loss of nuclear polarity with slight pseudostratification (black arrow). Scale bar, 25  µm. (**C**) *Cryptosporidium* developmental stages were observed in apical position (arrows) within the epithelial cells in a human colon adenocarcinoma of a Lebanese patient. Scale bar, 5  µm. (Hematoxyline and Eosine). Photomicrographs (**B**,**C**) were slightly modified from [41] and [40], respectively.

Several growing pieces of clinical evidence about links between cryptosporidiosis and human digestive neoplasia in different populations strengthen the idea of a possible causal relation. The association of cryptosporidiosis and colonic adenocarcinoma was evoked in the case of a Spanish patient presenting both, who died quickly after the onset of clinical manifestations [33]. A cryptosporidiosis case of the biliary tract clinically mimicking a pancreatic cancer in an HIV/Acquired Immunodeficiency Syndrome (AIDS) patient was also described [34]. Other studies reported elevated colon carcinoma risk in AIDS patients with cryptosporidiosis [35] and bile duct carcinoma associated with *Cryptosporidium* infection in children with X-linked hyper-IgM syndrome and in immunodeficient mice [36,37,38]. Authors of the latter studies proposed that the mutation responsible for this defect might favor the colonization of the biliary epithelium by *Cryptosporidium*. A chronic infection by this parasite will follow and inflammation may be the cause of the malignant transformation [37,38]. Even if this condition is mainly diagnosed in children, a case of an adult patient with CD40L deficiency who suffered of cholangiocarcinoma arising from sclerosing cholangitis associated with chronic cryptosporidiosis was also reported [39]. In parallel, a clinical study conducted in Lebanon strongly suggested evidence for a link between cryptosporidiosis and colorectal cancer. Indeed, *Cryptosporidium* infection was identified by PCR in 21% (15/72) of biopsies from patients with recently diagnosed colon neoplasia (including low- or high-grade intraepithelial neoplasia and not invasive/invasive adenocarcinoma) before any treatment compared to 7% (9/146) of biopsies from patients without digestive neoplasia but with persistent gastrointestinal symptoms. The risk of *Cryptosporidium* infection was thus 4 times higher in the first group. Moreover, the molecular characterization of the corresponding *Cryptosporidium* isolates allowed for the identification of either *C. parvum* or *C. hominis.* In addition, the presence of *Cryptosporidium* developmental stages in the apical position within the epithelial cells of the intestinal glands was confirmed [40] (Figure 3).

Consistently, various clinical studies conducted in Poland confirm these observations [42,43,44]. On the other hand, in another cohort of immunocompetent patients from Poland with colorectal cancer, the presence of *Cryptosporidium* was found in one patient out of 145. After genotyping, the presence of *C. meleagridis* was identified, this being the first study reporting an association of this *Cryptosporidium* species with colon adenocarcinoma [45]. More recently, a Chinese case control study described a *Cryptosporidium* infection rate of 17.24% (20/116) in patients with colorectal cancer before chemotherapy. In addition, the same authors reported the presence of the parasite in liver, esophageal and small intestine cancers [46]. A summary of different reports about the link between *Cryptosporidium* infection and human digestive neoplasia in different populations is shown in Table 1.

Besides direct clinical evidence suggesting the association between *Cryptosporidium* and digestive cancer development, some studies conveyed that the risk of developing colon carcinoma is significantly elevated among AIDS patients, a group at risk of *Cryptosporidium* infection [47]. However, *Cryptosporidium* is an opportunistic agent that causes important morbidity and mortality in persons with immunodeficiency. Therefore, it is possible that immunocompromised people have a higher risk of developing malignancy induced by this parasite, especially when their immunosuppression is more severe. In fact, *Cryptosporidium* infection has been suggested to be associated with some malignancies such as leukemia [48]. Nevertheless, a meta-analysis recently reported that, even if a positive association was found between *Cryptosporidium* infection and cancer in general (OR = 3.3; 95 CI: 2.18–4.98), the occurrence of *Cryptosporidium* infection was mainly related to colorectal cancer (OR = 3.7; 95 CI: 2.10–6.50) but not to other types of malignancies, such as blood cancer [49], suggesting that the parasite is not a special risk to cancer patients. Even if there are several clinical pieces of evidence from different geographical areas, the majority of the articles in the literature are case reports. Further prospective studies should be conducted based on clinical trials using sensitive diagnostic tools for the identification of the parasite.

### 4.2. Natural or Experimental Infection in Animals

Links between non-malignant tumors or atypical histology and *Cryptosporidium* were already reported in naturally or experimentally infected animals. For example, two studies described an association between aural or aural-pharyngeal polyps and *Cryptosporidium* infection in iguanas [50,51]. An intestinal metaplasia of the proventriculus was also associated with *Cryptosporidium baileyi* in a snowy owl [52]. Moreover, histological analysis of twenty-three leopard geckos revealed a *Cryptosporidium* sp. infection associated with hyperplasia of small and large intestine [53]. To complete this overview, low grade dysplasia in bile ducts has also been reported in an experimental model of IFN-δ knockout mice infected with *C. parvum* [37]. Nevertheless, no studies, to our knowledge, described an association between *Cryptosporidium* sp. infection and malignant tumors, until 2007 [54]. Indeed, in order to explore the dynamics of *Cryptosporidium* infection, an animal model of cryptosporidiosis was developed using corticoid dexamethasone-treated or untreated adult Severe Combined Immunodeficiency (SCID) mice, orally infected with *C. parvum* or *C. muris* oocysts. Intriguingly, only *C. parvum*-infected animals developed ileo-caecal adenocarcinoma as soon as 45 days post-infection even when mice were infected with only one oocyst (Figure 3A) [54,55]. The inoculation of animals was performed with the *C. parvum* Iowa strain isolated from cattle and which is the reference strain for this *Cryptosporidium* species (first *C. parvum* strain to have an entire genome sequenced). However, additional *C. parvum* strains were tested in the same murine model including “TUM1” (isolated from a calf in the USA), and “Did” and “CHR” (isolated from patients in the Centre Hospitalier Universitaire de Lille, France). These three isolates were found to be more virulent than the Iowa strain [56,57,58]. Indeed, they induced a higher mortality rate, an earlier onset of neoplastic lesions (as soon as 15 days post-infection for the CHR strain), and a more rapid progression to invasive adenocarcinoma. Interestingly, the development of intestinal low- and high-grade dysplasia after only 30 days post-infection with *C. parvum* in a model of dexamethasone treated immunocompetent Swiss albino mice was reported by others [59]. However, adenocarcinoma development was not detected in this model, probably due to the early time of euthanasia. Similar observations were reported by others using the same mice model [60]. Moreover, the severity of the dysplastic lesions seemed to be correlated with the intensity of oocyst shedding [61].

### 4.3. In Vitro Models

Recently, a three-dimensional (3D) in-vivo-like culture model from adult murine colon was developed, allowing for biological investigations of *Cryptosporidium* infection and a better study of its pathophysiology. Indeed, the resulting system allowed for the maintaining of the infection but also the development of low-grade intra-epithelial neoplasia in vitro after only 27 days post-infection (Figure 3B) [41]. This model is currently adapted to human intestinal tissue in order to look for further evidence on the role of *C. parvum* and/or *C. hominis* in the induction of human colon cancer.

## 5. Hypotheses about Molecules and Mechanisms Involved in the Induction of Tumorigenesis by *Cryptosporidium*

**Figure 4:**
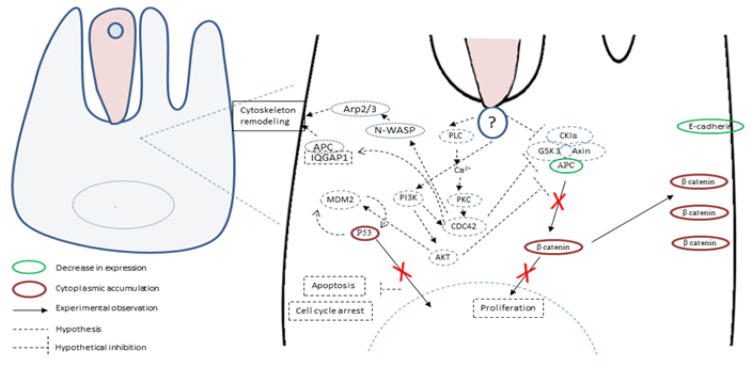
Hypothetical host-cell signaling pathways linking *Cryptosporidium parvum* infection and tumorigenesis. Different studies have shown that *C. parvum* induces cytoskeleton remodeling and actin reorganization through the implication of several intracellular signals, including phosphatidylinositol 3-kinase (PI3K) and Cdc42. In addition, in an experimental model, Adenomatous Polyposis Coli (APC) and E-cadherin labelings were decreased while β-catenin and the P53 tumor suppressor labelings were increased in the cytoplasm of *C. parvum-*infected epithelial cells. β-catenin was found localized in a juxtamembraneous position, suggesting a role of the non-canonical Wnt pathway in this transformation process. P53 was not translocated into the nucleus, and its labeling increased in the cytoplasm where P53 could regulate key metabolic pathways associated to apoptosis and cell cycle arrest.

The pathophysiological mechanisms of *Cryptosporidium* infection are multifactorial and not completely understood. Some advances were achieved recently and revealed that the infection by *C. parvum* induces cytoskeleton remodeling and actin reorganization through the implication of several intracellular signals involving, for example, PI3K, Src, Cdc42 and GTPase [62,63] (Figure 4).

Consistently, signal transduction pathways targeting cell proliferation, cellular junctions and adhesions have also been described in gastric cancer induced by *H. pylori* [64]. It was also reported that the infection by *C. parvum* leads to the activation of NF-κB [62], known to induce anti-apoptotic mechanisms and also to transmit oncogenic signals to epithelial cells [65,66]. In addition, microarray assays were recently performed on *C. parvum* IId Human Ileocaecal Adenocarcinoma (HCT-8)-infected cells. A differential profile of mRNAs was found between infected and non-infected cells. Indeed, mRNAs of the Wnt and hedgehog signaling pathways were significantly differentially expressed in infected cells compared to not infected ones [67]. Noticeably, these two pathways are also involved in the development and progression of colorectal cancer [68]. Despite the growing evidence about the hijacking of cellular pathways potentially being involved in cancer onset, this information has rarely been linked to the tumorigenic potential of the parasite. To our knowledge, only one study tried to decipher this process and highlighted the important role of the Wnt signaling pathway and the alteration of the cytoskeleton in the carcinogenic process induced by *C. parvum* experimental infection [69]. Indeed, the immunohistochemical analysis of ileocecal region sections embedded in paraffin from *C. parvum* infected *versus* non-infected mice showed alterations in APC, β-catenin, P53 and E-cadherin expression [69]. APC and E-cadherin labelings were decreased while those of β-catenin and P53 were increased in the cytoplasm of epithelial cells. In addition, the immunofluorescence analysis of these histological sections confirmed a membranous and juxtamembraneous localization of β-catenin without nucleus translocation, suggesting an involvement of the non-canonical Wnt pathway [69]. However, unlike *Helicobacter pylori*, for which bacterial virulence factors associated to the gastric cancer outcome were identified, such as cytotoxin associated gene A (CagA), vacuolating cytotoxin A (VacA) and outer inflammatory protein A (OipA) [70], virulence factors implied in the *C. parvum*-induced carcinogenic process remain unknown. The whole genome sequences of different species and isolates of *Cryptosporidium* are now available [71,72,73]. Hence, a comparative genomic analysis of different *C. parvum* strains (Did, TUM1, CHR and Iowa, the reference strain) with variable virulence was recently performed [58]. Overall, 125 common SNVs (single nucleotide variation) (corresponding to 90 CDSs (coding sequences) were found in the three more virulent strains (Did, TUM1 and CHR) compared to Iowa strain. The majority of these genes are over-expressed in the intracellular stages of the parasite. In addition, mucins, transporters (ABC and ATPase3) and cysteine proteases were also found, these genes being already described as virulence factors. This study also reported new potential factors involved in the virulence of *C. parvum* such as various phosphatases (PP2A, Cdc14) and a histone-lysine *N*-methyltransferase. Further investigations are needed to elucidate carcinogenic mechanisms induced by *C. parvum*. In particular, the biological function study of the potential virulent and/or carcinogenic factors identified could be facilitated by the use of genome-editing tools such as CRISPR/Cas9 [58].

## 6. Conclusions and Future Directions

Is colon cancer a cause or a consequence of *Cyptosporidium* infection? We presented an updated picture of the link between digestive cancer and infection by this parasite. Available experimental and clinical data synthesized herein suggest that the parasite is able to employ strategies to target cellular processes during its complex interactions with host cells, leading to a parasite-induced transformation. However, the fact that Cryptosporidium is an opportunistic agent is one of the main difficulties for proving its role in the induction of human digestive cancers. In addition, available experimental models with little or no immune response are not necessarily reflecting the human condition. Despite several pieces of evidence about associations between cryptosporidiosis and digestive neoplasia, it seems that not enough attention has been paid to the tumorigenic power of this protozoan parasite. Therefore, it is necessary to demonstrate a direct causal link, and to identify virulent factors and carcinogenic mechanisms responsible for the epithelial cell transformation. A potential solution would be to combine recent advances in 3D culture models, comparative genomic studies and transfection methods (CRISPR/Cas9 system) together with further clinical trials using sensitive diagnostic tools. If the causal link between *Cryptosporidium* and human cancer is clearly established, a great number of digestive cancers could be prevented using public health measures to reduce the risk of *Cryptosporidium* infection. In addition, this could entice researchers to explore new therapeutic targets and vaccines in order to clear or prevent the infection, thereby saving hundreds of thousand children from severe diarrhoea and mortality [24]. Research into this topic is urgently needed since the incidence of *Cryptosporidium* infection is increasing worldwide.

## 7. Search Strategy and Selection Criteria

We selected articles from PubMed and Google Scholar using the research terms “*Cryptosporidium*”, “cancer”, “oncogenesis”, “infection and cancer”, “colorectal cancer”, “cryptosporidiosis”, “parasites and cancer”, “*Cryptosporidium* and cancer”, “cancer causes”, “epigenetics and cancer”, “*Cryptosporidium* and virulence factors”, “digestive neoplasia”, “colon adenocarcinoma”, “cancer and mechanisms”. Reviews, clinical, epidemiological and experimental data were taking into account without restriction of date or language. Preference was given to the articles published within the past 20 years.

## Figures and Tables

**Table 1:** Associations between *Cryptosporidium* infection and human digestive neoplasia in different populations.

Type of Cancer	Type of Study	Geographic Localization	Clinical Sample:Laboratory Method	*n* (%)	*p*-Value	Immuno-Suppression	Reference
Colonic adenocarcinoma	Case report	Spain	Not reported	1	NA ^a^	No	[33]
Pancreatic cancer	Case report	Brazil	Tissues/Microscopical observation	1	NA ^a^	HIV/AIDS	[34]
Colorectal cancer(Adenocarcinoma)	Data matching between HIV/AIDS and cancer registry databases in 16 U.S. states	United States	Tissues/Microscopical observation	3/269 (1%)	0.70	HIV/AIDS	[35]
Colorectalsquamous cell carcinoma	Data matching between HIV/AIDS and cancer registry databases in 16 U.S. states	United States	Tissues/Microscopical observation	1/8 (12.5%)	0.02 ^b^	HIV/AIDS	[35]
Uncommon colorectal cancers)	Data matching between HIV/AIDS and cancer registry databases in 16 U.S. states	United States	Tissues/Microscopical observation	3/43 (7%)	0.04 ^b^	HIV/AIDS	[35]
Bile duct carcinoma	Case reports	United States	Tissues/Microscopical observation	Not reported	NA ^a^	X linked immunodeficiency with hyper-lgM	[36]
Hepatoma	Analysis of the USIDNET Registry	United States	Not reported	1/145(1%)	NA ^a^	X-linked hyper-IgMsyndrome in children	[38]
Cholangiocarcinoma	Case report	United Kingdom	Stool samples,Coprological analysis	1	NA ^a^	CD40L deficiency	[39]
Colonic adenocarcinoma	Case-control	Lebanon	DNA from biopsies, PCR	15/72 (21%)	0.003 ^b^	No	[42]
Colonic adenocarcinoma	Cases	Poland	Stool samples, coprology and ELISA	4/55(18%)	NA ^a^	No	[43]
Colonic adenocarcinoma	Cases	Poland	Stool samples,ELISA	10/87(12%)	NA ^a^	No	[44]
Colonic adenocarcinoma	Case-control	Poland	Stool samples, coprology analysis and ELISA	14/108 ^b^(13%)	0.015 ^b^	No	[45]
Colonic adenocarcinoma	Cases	Poland	DNA from stools, PCR	1/145(1%)	NA ^a^	No	[46]
Colonic adenocarcinoma	Case-control	China	DNA from stools, PCR	20/116 (17.24%)	<0.001 ^b^	No	[46]
Gastric	Case-control	China	DNA from stools, PCR	2/51 (4%)	0.121	No	[46]
Esophageal	Case-control	China	DNA from stools, PCR	1/16 (6.25%)	0.029 ^b^	No	[46]
Liver	Case-control	China	DNA from stools, PCR	1/7 (14.29%)	<0.001 ^b^	No	[46]
Small Intestine	Case-control	China	DNA from stools, PCR	2/5 (40%)	<0.001 ^b^	NO	[46]

^a^ NA, Not applicable. ^b^ The difference between the prevalence of the cases and controls is statistically significant.
